# Electro-hydrodynamic programming reshapes liquid crystal dynamics in free-form director fields

**DOI:** 10.1038/s41598-024-54873-5

**Published:** 2024-02-20

**Authors:** Vinayak Ghorapade, Wei-Chih Wang

**Affiliations:** 1 Institute of NanoEngineering and MicroSystems, National Tsing Hua University, Hsinchu, 30013 Taiwan, ROC; 2 Department of Power Mechanical Engineering, National Tsing Hua University, Hsinchu, 30013 Taiwan, ROC; 3 Department of Electrical and Computer Engineering, University of Washington, Seattle Washington, 98195 USA; 4 Department of Mechanical Engineering, University of Washington, Seattle Washington, 98195 USA

**Keywords:** Engineering, Materials science, Mathematics and computing, Nanoscience and technology, Optics and photonics

## Abstract

This study unveils a groundbreaking technique leveraging the superposition of electric field vectors to manipulate liquid crystals (LCs). Demonstrated through a simple configuration of four independent electrodes at the corners of a rectangular enclosure, notably, this configuration can be further simplified or modified as needed, showcasing the versatility of the approach. Significantly, the design showcased in the paper eliminates the need for an alignment layer, highlighting the versatility of the method. Through nuanced adjustments in waveforms, amplitudes, frequencies, and phases in AC or DC from these electrodes, precise control over LC shape deformation and dynamic phase transformation is achieved in both temporal and spatial dimensions. In contrast to traditional methods, the approach presented here abolishes alignment layers and intricate electrode-array systems, opting for a streamlined configuration with varying AC frequencies and DC electric signals. This innovative methodology, founded on simplified governing equations from Q-tensor hydrodynamics theory, demonstrates true 3D control over LCs, displaying efficiency in electrode usage beyond current arrays. The study's contributions extend to temporal control emphasis, superposition techniques, and the elimination of fixed electrodes, promising unprecedented possibilities for programming LC materials and advancing the field of programmable LC devices.

## Introduction

Nematic liquid crystals (LCs) have numerous potential applications in the electro-optic field due to their anisotropic optical properties. They have been used as spatial light modulators, compact spectrometers^1^, terahertz filters^2^, and more. Additionally, liquid crystals have many applications in the medical field for optical diagnosis and biosensors^3^. However, Most of the previous liquid crystal research is limited to the liquid crystal cell structure with alignment layers and one or two-directional electric fields with electrodes in a single plane or with multiple electrode arrays.

Several advanced reconfigurable liquid crystal-based devices have been developed, including fast-reconfigurable phase shifters, devices for generating optical vortices, LC-based reconfigurable intelligent surfaces, and liquid crystal gratings. For example, Kim et al. designed a fast-reconfigurable phase shifter utilizing liquid crystal, evaluating its performance for twisted nematic and antiparallel alignment liquid crystal cell configurations^4^. Albero et al. developed two reconfigurable LC devices to generate optical vortices. The first device featured a 12-slice pie-shaped electrode with vertical alignment, creating a spiral phase plate (SPP). This SPP, combined with two-quarter plates, functioned as a pseudo-radically polarized beam. Their second device utilized a spiral electrode shape, acting as a spiral diffractive lens^5^. Meng et al. constructed an LC-based reconfigurable intelligent surface (RIS) with unit cells filled with LC between the antenna patch and ground, forming a reflect-array to control beam scanning^6^. Shen et al. simulated and characterized a reconfigurable liquid crystal grating based on the Oseen Frank theory^7^. Foo et al. developed a metasurface reflector utilizing reconfigurable liquid crystal cells, controlled by individual microstrip patches on each unit cell^8^. Similarly, You et al. created a reconfigurable periodic liquid crystal array to induce topological defects in each unit cell by controlling electric fields, observing changes in patterns^9^.

Despite these seemingly innovative designs, these advanced reconfigurable systems are constrained in their tunability, relying on planar electrode structures, fixed mechanical alignment layers which limit the orientation of the liquid crystal director to a finite set of directions, and array configurations which only allow them to generate a planar distribution of LC orientation. Many of these designs are extremely complicated, presenting challenges in fabrication and operational processes. To address these limitations, we developed a new technique that manipulates liquid crystal molecules using the superposition of electric field vectors, enhancing programmability by eliminating the need for alignment layers. Unlike current reconfigurable devices, which rely on alignment layers and fixed electrode configurations, our approach eliminates the need for alignment layers and allows for independent control over liquid crystal orientation in any direction. This flexibility is achieved through the utilization of the superposition of electric fields with a four-electrode configuration around four corners of a rectangular enclosure. Notably, this configuration can be further simplified or modified as needed, showcasing the versatility of the approach. Our approach enables liquid crystal directors to reorient in any direction. By manipulating waveforms, amplitudes, frequencies, and phases in AC or DC from these electrodes, applied bias voltages impose temporary constraints, allowing for dynamic changes in optical structures. We achieve precise control over the shape deformation and dynamic phase transformation of LCs in both temporal and spatial domains, enabling precise control over the liquid crystal director's orientation in three-dimensional space and time.

While previous studies, such as the one by Izdebskaya et al. have explored liquid crystal manipulation on multifunctional metasurface tuning without alignment layers and controlling liquid crystal director orientation by rotating the direction of an applied external magnetic field^10^. However, this magnetic alignment primarily tunes the optical properties of a layer above the metasurface in three-dimensional space. These approaches still lack the independent control over individual liquid crystal orientations that our technique offers. In contrast, our research enables three-dimensional control of liquid crystal director orientation over both space and time, resulting in diverse birefringence patterns.

In the following section, we will first introduce the concept by modeling programmable LC devices based on simplified governing equations derived from Q-tensor hydrodynamics theory. For the demonstration, we will illustrate how, by manipulating waveforms, amplitudes, frequencies, and phases in AC or DC from some simple surrounding electrodes, we achieved precise control over the shape deformation and dynamic phase transformation of LCs in both temporal and spatial domains.

**Figure 1:**
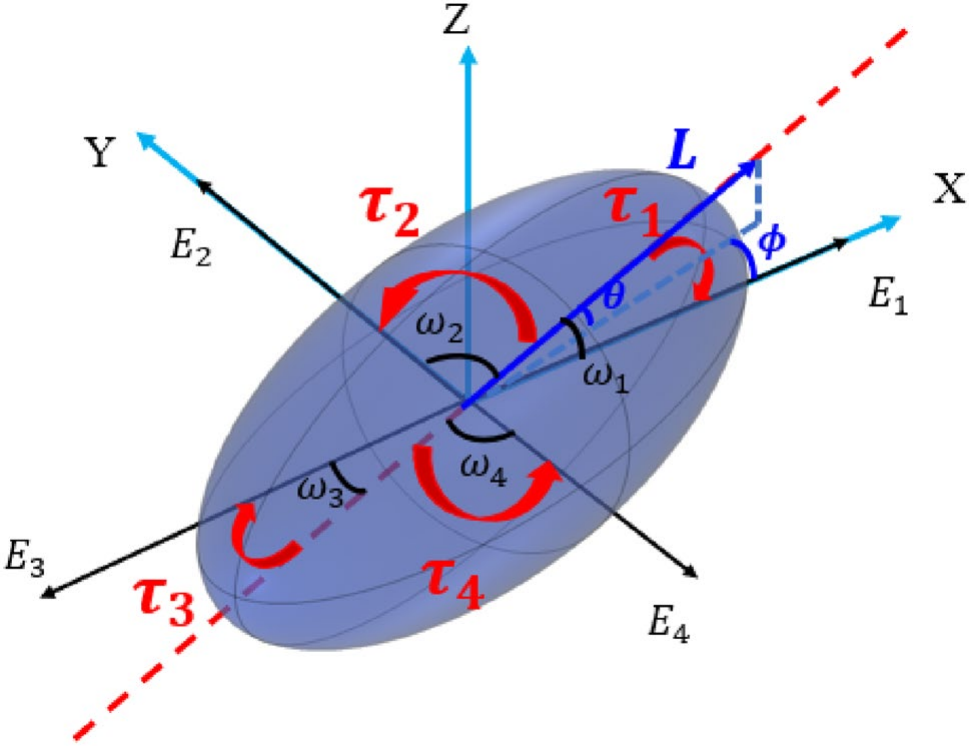
Liquid crystals molecule experiencing three-dimensional torques due to the applied electric field in the four-electrode system.

In this study, we are focused on very important and significant part of the hydrodynamics i.e. rotational orientation of the liquid crystal molecules in which the flow of liquid crystal molecules is insignificant. To create a single domain system with ease of design and operations for programmable liquid crystal, we are proposing a novel technique with three-dimensional control and manipulation of liquid crystal molecules in space and time using superposition of electric field vectors in a multiple electrode device. This novel technique involving the superposition of electric field vectors is utilized to manipulate and control the liquid crystal molecules without the need for alignment layers. These manipulations induce splay, bend, and twist deformations in the liquid crystals. The superposition of electric fields from the four electrodes creates local torques on the liquid crystal molecules in space and time, as depicted in Fig. 1. The balancing of these torques results in three-dimensional splay, bend, and twist deformations, allowing for localized control of liquid crystal orientation and the generation of diverse anisotropic properties. This level of control and dynamic manipulation is not achievable with conventional two-electrode configurations with fixed mechanical boundaries, and also in recent advanced reconfigurable liquid crystal devices. The driving and bias voltages can be either constant or variable. In this study, the bias voltage is kept constant to maintain the molecules in one plane while changing the director orientation in the other two planes.

The external electric energy generates volumetric torques (τ) in the liquid crystals, which attempt to align the director of the liquid crystal with the electric field E^11^. In this study, the superposition of electric fields creates multiple torques on the liquid crystal molecules, which vary in space and time. As a result, the torques in the simulated model are localized, acting on specific regions of the liquid crystals, unlike conventional liquid crystal devices where volumetric torques act uniformly throughout the device. The local action of volumetric torques due to the electric fields ${E}_{1}, {E}_{2}, {E}_{3}$ and ${E}_{4}$ in the four-electrode system is,

1 $${\tau }_{i}={\varepsilon }_{0}\Delta \varepsilon {{E}_{i}}^{2}{\text{sin}}\left({\omega }_{i}\right){\text{cos}}({\omega }_{i})$$

where ${\omega }_{i}$ is the angle between the extraordinary axis of liquid crystals and the electric field vector ${E}_{i}$. Change in the angle ${\omega }_{i}$ during the alignment due to torques results in the change of polar angle $(\theta$) and azimuth angle ($\phi$) of the directors of the liquid crystal.

In a multi-electrode system, the electric field in a plane creates a symmetric electric field near the midpoint of the electrodes. This symmetry can be defined using the proper sign convention in all three directions using a unit vector ${\widehat{E}}_{i}$ as given by Eq. (2). These sign conventions in respective directions will act as reference directions for the director orientations.

2 $${\widehat{E}}_{i}=\frac{\overline{{E }_{i}}}{\left|\overline{{E }_{i}}\right|}$$

where, $\overline{E }$ is the electric field vector and $i=x, \,y\, and\, z$. This unit vector ${\widehat{E}}_{i}$ is used as the multiplier for the director fields in each direction.

The most crucial step in liquid crystal simulations is computing the orientation of the liquid crystal molecules against the controlling vectors and then calculating the properties based on the orientation field, also known as the director field. There are two continuum theories for computing the molecular orientations of liquid crystals namely the Landau-de Gennes Q tensor theory and the Oseen-Frank theory. Frank and Oseen made significant contributions to the initial work^12,13^. Frank developed the basic mathematics describing the elasticity of liquid crystals, while Oseen described the generalized equations for the molecular model based on the continuum theory that enables the static formulation of liquid crystal molecular orientation. Later, de Gennes^14,15^ defined the free energy for liquid crystals in scalar form using the invariant in ordering tensor form. This order parameter, also known as the director, provides information on phase transformation. Subsequently, Ericksen-Leslie^16,17^ and Qian-Sheng^18^ extended the Oseen-Frank and Landau-de Gennes theories, respectively, and introduced the viscous stress that enables dynamics in the computation of the orientation of liquid crystals, as discussed later in the Methodology section.

Nematic liquid crystals molecules are approximated as rigid rods with both ends indistinguishable from the one other defined by a unit vector $l$^19^, as given in the Eq. (3).

3 $$l=-l=l$$

For random order by thermal averages gets cancelled,

4 $$<{l}_{i}>=<{l}_{i}{l}_{j}{l}_{k}>= ... =0$$

Optically, these are uniaxial materials and the magnetic susceptibility is also of the uniaxial form. Considering Eq. (3), it can be stated that,

5 $$<{l}_{i}{l}_{j}>=S*{n}_{i}{n}_{j}+\frac{1}{3}\left(1-S\right){\delta }_{ij}$$

where n, called the director, is a unit vector parallel to the optic axis and $S$ is the scalar order parameter, $\delta$ is the Kronecker delta^20^.

For random and perfect alignment, $S$ = 0 and $S$ = 1 respectively. However, the alignment usually is not perfect for the nematic LCs. In addition, the $S$ parameter changes with the temperature; experimental values vary from 0.3 to 0.7^20^.

**Figure 2:**
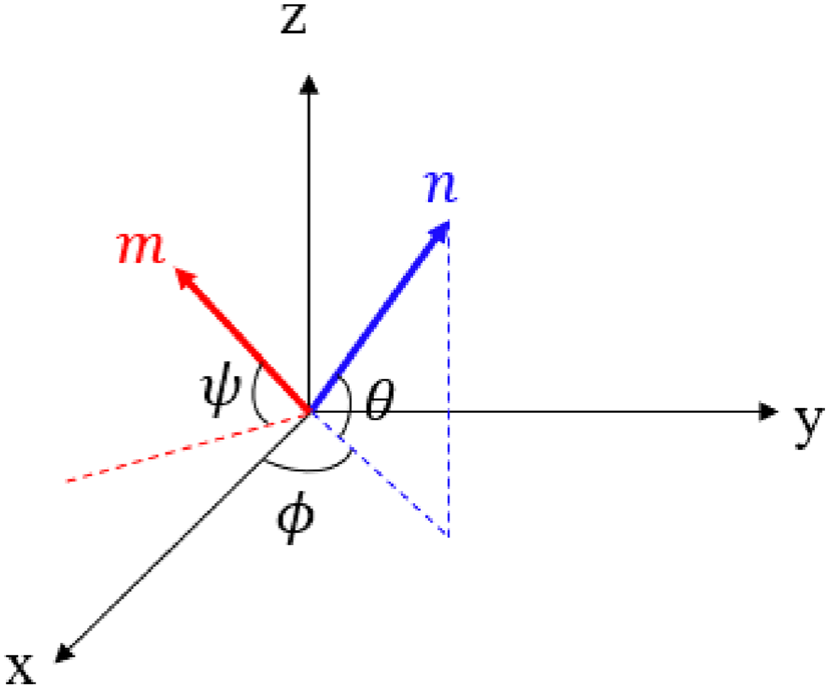
The orientation of director vectors n and m using Euler’s angles $\theta$, $\phi$ and $\psi$ in X, Y and Z laboratory axes.

At any local position in the liquid crystals, the average orientation change of the molecules is given by an order parameter called the director^21^. In general, the biaxial nature of nematic liquid crystals is defined by two orthonormal eigenvectors (n and m) and two scalar order parameters (S and P) as shown in the Fig. 2.

The director vectors $n$ and $m$ can be defined in terms of Euler angles $\theta$, $\phi ,$ and $\psi$ as follows,

6 $$\left.\begin{array}{c}n=\left[{\text{cos}}\left(\phi \right){\text{cos}}\left(\theta \right);{\text{cos}}\left(\theta \right){\text{sin}}\left(\phi \right);{\text{sin}}\left(\theta \right)\right]\\ m=[sin(\phi ) cos(\psi )-cos(\phi ) sin(\psi ) sin(\theta );\\ -{\text{sin}}\left(\phi \right){\text{sin}}\left(\psi \right){\text{sin}}\left(\theta \right)-cos\left(\phi \right)cos\left(\psi \right);\\ sin(\psi )cos(\theta )]\end{array}\right\}$$

For biaxial nematic liquid crystals, Q-tensor is represented in terms of director vectors $n$ and $m$ as,

7 $${Q}_{ij}=S\left({n}_{i}{n}_{j}-\frac{1}{3}{\delta }_{ij}\right)+P({m}_{i}{m}_{j})$$

where scalar order parameters $S$ and $P$ give the quantitative values of the degree of orientation for the liquid crystals molecules and ${\delta }_{ij}$, justifies the traceless and symmetric nature of the Q (3 × 3) matrix.

E. Willman^22^ implemented Q-tensor hydrodynamics for finite element analysis to simulate simple geometries. The Q-tensor is a representation of all the director positions in a single matrix, which has rank two and three eigenvalues (determined by two independent variables) corresponding to orthonormal eigenvectors n and m (Eq. (7)). The purpose of using a Q-tensor is for energy minimization. In this case, the Q-tensor is given in the following form:

8 $$Q=\left[\begin{array}{ccc}{q}_{11}& {q}_{12}& {q}_{13}\\ {q}_{21}& {q}_{22}& {q}_{23}\\ {q}_{31}& {q}_{32}& {q}_{33}\end{array}\right]$$

where $${q}_{33}=-{q}_{11}-{q}_{22}$$.

In K.R. Daly's study^23^, an algorithm was developed for fast and accurate computation of liquid crystal hydrodynamics using Q-tensor theory to simulate a two-dimensional LC model based on the Q-tensor. In this study, we formulated five governing equations using five auxiliary variables derived from the Q-tensor (3 × 3) governing differential equation, considering the symmetric and traceless nature of the Q-tensor. These five equations, along with the pre-solved electrostatic field, compute the orientation of the liquid crystal molecules in response to various internal and external forces. These equilibrium orientations at different time steps are then used to determine the anisotropic properties of the liquid crystals.

The multiple electrode system creates a superposition of electric fields from these electrodes. The set of governing equations formulated allows for the demonstration of the effect of superimposed electric field vectors on liquid crystal molecules. The torques generated by the interaction of electric field vectors and liquid crystal molecules provide three-dimensional control over the orientation of the liquid crystal molecules, eliminating the need for an alignment layer as required in conventional liquid crystal cells. This three-dimensional torque-based control enables the creation of more arbitrary patterns of liquid crystal molecule orientations beyond simple fixed patterns achievable with an alignment layer. This allows electrodynamic programming for different patterns of liquid crystal molecule orientations in three dimensions, leading to optical birefringence of the liquid crystal.

**Figure 3:**
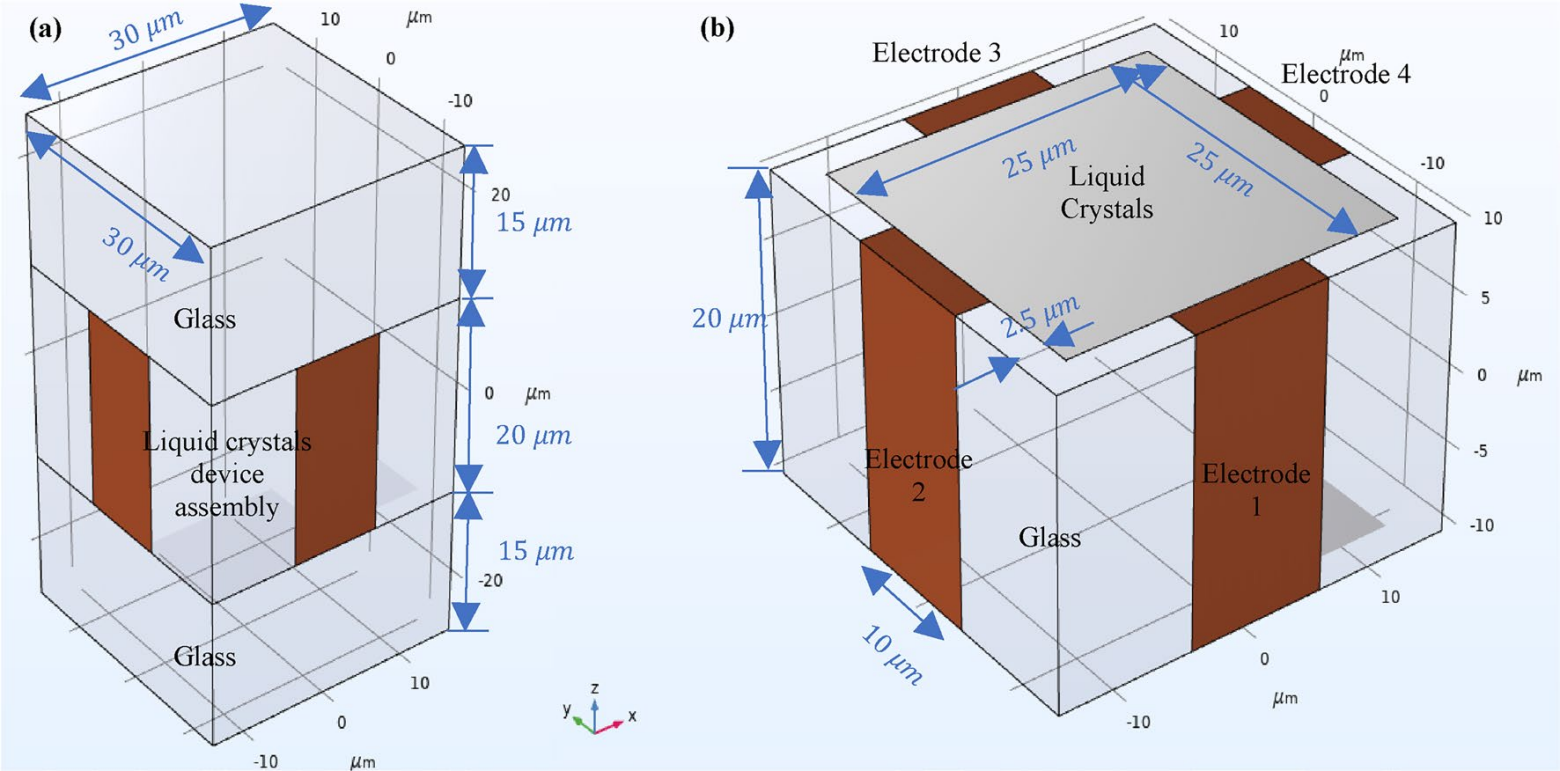
Schematic of Liquid crystal device. (**a**) LC device with top and bottom substrate sandwiching LC cell assembly (**b**) Detailed view of LC cell assembly.

To illustrate this concept, we simulate a simple design using a rectangular enclosure with four electrodes placed on four out of six sides (Fig. 3). Here, different orientation holding methods, voltages, and waveforms can be input to control shape deformation and dynamic phase transformation in time and space in the liquid crystals. Furthermore, a wide range of interesting time- and space-varying patterns of the liquid crystal's director field can be generated by combinations of different AC and DC voltages. In this paper, we also demonstrate some interesting twisting structures that vary in space and time. The twisting can be controlled in the twist direction, and different twist structures can be created in the perpendicular direction to the twist direction, such as a gradient.

This electrodynamic programming of liquid crystal materials will enable one to advance liquid crystal applications in more complex and three-dimensional systems beyond the current use in flat panel LCDs and other simple applications. By varying design parameters such as input energy, signal combinations, electrode numbers, size and shape of electrodes, and patterns of electrodes, we can create simple or complex cell structures that generate a wide range of anisotropic patterns. This can include mimicking different shapes of optical components with varying functionality, generating structures with freedom to create different domains with various molecular orientations, and achieving uniaxial, biaxial, or anomalous birefringence by assembling small domains to form patterns with different anisotropic properties. This feature can even be further utilized to create new and complex electromagnetic structures with new degrees of freedom in designs and operations.

## Methodology

**Figure 4:**
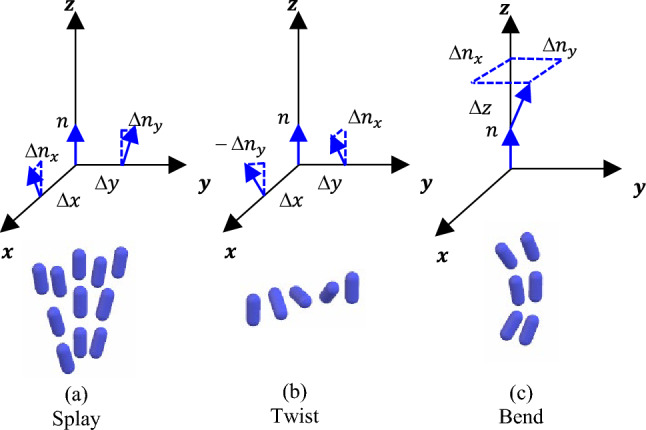
Types of deformation in nematic liquid crystal (**a**) Splay deformation of the liquid crystal (**b**) twist deformation of the liquid crystal **c** bend deformation of the liquid crystal.

In this simulation, we consider elastic distortion energy, Landau-de Gennes bulk free energy, viscous energy, and external interactions, such as external electric energy. These energies are defined in tensor form and in this study; they are converted into a set of governing equations with energy balancing using auxiliary variables. For the bulk nematic director field, there are three possible deformations, namely, splay, twist, and bend distortions, as shown in Fig. 4.

### Continuum theory

The free energy density is a thermodynamic potential, the minimization of free energy density gets the system to stable or metastable state^24^. The differentiation of initial internal energy density interactions in the liquid crystals can be governed using equation^25^,

9 $${\text{f}}=-\frac{\partial \mathrm{\varnothing }({\text{Q}})}{\partial {{\text{Q}}}_{{\text{ij}}}}+\frac{\partial {{\text{f}}}_{{\text{d}}}}{\partial {{\text{Q}}}_{{\text{ij}}}}$$

where, ${{\text{f}}}_{{\text{d}}}$ is the elastic distortion energy density, $\mathrm{\varnothing }\left({\text{Q}}\right)$ is the thermotropic (or Landau) energy density. These energies are minimized using Euler–Lagrange’s formulation for the initial equilibrium state.

For the elastic distortion energy density, fd is written as a function of the director and its spatial derivatives. For nematic liquid crystals, this term is minimized when the director field is in an undistorted configuration. For chiral LCs, the minimum occurs when a twist deformation with a pitch length of $p$ is present. The elastic energy density can also be expressed in terms of the Q-tensor and its spatial derivatives as:

10 $${f}_{d}=\frac{1}{2}{L}_{1}{Q}_{ij,k}{Q}_{ij,k}+\frac{1}{2}{L}_{2} {Q}_{ij,j}{Q}_{ik,k}+\frac{1}{2}{L}_{3} {Q}_{ik,j}{Q}_{ij,k} +\frac{1}{2}{L}_{4} {\epsilon }_{lik}{Q}_{lj}{Q}_{ij,k}+\frac{1}{2}{L}_{6} {Q}_{lk}{Q}_{ij,l}{Q}_{ij,k}$$

$${L}_{1}=\frac{1}{27{S}_{0}^{2}}\left({K}_{33}-{K}_{11}{+3K}_{22}\right), {L}_{2}=\frac{1}{9{S}_{0}^{2}}\left({K}_{11}-{K}_{22}{-K}_{24}\right); {L}_{3}=\frac{1}{9{S}_{0}^{2}}\left({K}_{24}\right); {L}_{4}=\frac{8\pi }{{p}_{0}9{S}_{0}^{2}}{K}_{22}$$

$${L}_{6}=\frac{2}{27{S}_{0}^{3}}({K}_{33}-{K}_{11})$$

The single elastic coefficients simplification (i.e. ${K}_{11}={K}_{22}={K}_{33}=K$ and ${K}_{24}=0$) in terms of Q is,

11 $${f}_{d}\approx \frac{1}{2}{L}_{1}{Q}_{ij,k}{Q}_{ij,k}$$

The thermotropic energy density (Landau de Gennes bulk free energy) is given by,

12 $$\mathrm{\varnothing }(Q)=\frac{1}{2}A{Q}_{ij}{Q}_{ji}+\frac{1}{3}B{Q}_{ij}{Q}_{jk}{Q}_{ki}+\frac{1}{4}C{{(Q}_{ij}{Q}_{ji})}^{2}$$

where $A$, $B,$ and $C$ are material parameters dependent on the temperature.

The generalized viscous energy density^18^ for the incompressible liquid crystals is given as

13 $${F}_{v}={\upbeta }_{1}{Q}_{ij}{Q}_{kl}{A}_{kl}+{\beta }_{4}{A}_{ij}+{\beta }_{5}{Q}_{ik}{A}_{kj}+{\beta }_{6}{Q}_{jk}{A}_{ki}+\frac{1}{2}{\gamma }_{2}{N}_{ij}-{\gamma }_{1}{Q}_{ik}{N}_{kj}+{\gamma }_{1}{Q}_{jk}{N}_{ki}$$

From D. Svensek and S. Zumer^25^ the viscous stress is given by,

14 $${-f}_{v}=\frac{1}{2}{\gamma }_{2}{A}_{ij}+{\gamma }_{1}{N}_{ij}=\frac{1}{2}{\gamma }_{2}{A}_{ij}+{\gamma }_{1}\left(\frac{\partial {Q}_{ij}}{\partial t}+({W}_{ik}{Q}_{kj}-{Q}_{ik}{W}_{kj})\right)$$

where, ${\upbeta }_{1}$, ${\upbeta }_{4}$, ${\upbeta }_{5}$, ${\upbeta }_{6}$,${\gamma }_{1}$ and ${\gamma }_{2}$ are the viscosity coefficients of the liquid crystals, ${A}_{ij}$ and ${W}_{ij}$ are the symmetric and antisymmetric parts of the velocity gradients. Here the approximations are made by excluding the quadratic flow terms from the equation, and limiting equation to uniaxial and flowless, gives the final viscous stress as,

15 $${-f}_{v}={\gamma }_{1}\left(\frac{\partial {Q}_{ij}}{\partial t}\right)$$

When external energies are applied, the director will try to align with these applied external energies such as solid surface and or electric field. The aligning effect of external electric fields or solid surfaces can be included by introducing energy density terms accounting for them. The effect of an external electric field can be expressed by writing the electric field energy density ${(f}_{f})$ in the usual way for a dielectric material^22^.

The applied electric field energy on a liquid crystal molecule is given as,

16 $${f}_{f}=\frac{1}{2}*D*E=\frac{1}{2}*{\varepsilon }_{0}*\overline{\overline{\varepsilon }}*\overline{E }.\overline{E }$$

where E is the electric field and D is the dielectric displacement. The effective electric permittivity is given as,

17 $$\overline{\overline{\varepsilon }}={{\varepsilon }_{\perp }\delta }_{ij}+\Delta \varepsilon \left(\frac{2}{3S}{Q}_{ij}+\frac{1}{3}{\delta }_{ij}\right),$$

where, ${\varepsilon }_{\perp }$ is the electric permittivity of LC molecules along the minor axis of the molecule and $\Delta \varepsilon$ dielectric anisotropy.

### Dynamic equilibrium Q-tensor fields and auxiliary equations

The free energy in the liquid crystal is the combination of elastic energy and Landau de Gennes bulk free energy in the minimized form. James R et al.^22^ in their study use summation of minimized free energy and electric energy. D. Svensek and S. Zumer^25^, use summation of minimized free energy and viscous energy density to study hydrodynamics of the liquid crystal. When an external energy like an electric field interacts with free energy density, the resulting dynamics are balanced by the viscous energy^18,22,25^,

18 $$F=f- {f}_{v}-\frac{\partial {f}_{f}}{\partial {Q}_{ij}} =0$$

19 $${\gamma }_{1}\frac{\partial {Q}_{ij}}{\partial t}=L{\partial }^{2}{Q}_{ij}-A{Q}_{ij}-B{Q}_{ik}{Q}_{kj}-C{Q}_{ij}\left({Q}_{kl}{Q}_{lk}\right)-\frac{1}{2}*{\varepsilon }_{0}*\Delta \varepsilon \left(\frac{2}{3S}{Q}_{ij}\right)*\overline{E }.\overline{E }\, in\,\Omega $$

where, ${\gamma }_{1}$ is the rotational viscosity of the liquid crystals and $\Omega$ representing the domain in the finite element analysis.

These governing equations in the domain and on the boundary are converted into the auxiliary form of governing equations, where Q-tensor in auxiliary form appears as,

20 $$Q=\left[\begin{array}{ccc}\frac{2\eta }{3}& \upsilon & \alpha \\ \upsilon & -\frac{1}{3}\eta +\mu & \beta \\ \alpha & \beta & -\frac{1}{3}\eta -\mu \end{array}\right]$$

This auxiliary form of Q tensor is symmetric and traceless and the behavior of Q tensor with respect to change in the energies can be defined by five governing equations, where $\eta$, $\mu$, $\upsilon$, $\alpha$ and $\beta$ are the auxiliary variables.

The governing equation (Eq. (19)) is reduced to a system of five equations, involving space and time differentials of the auxiliary variables. This system of equations balances the viscous, elastic, thermotropic, and external electric energies by updating the director orientation in space and time. The simulation involves two steps. First, we introduce the external electric force and differentiate the governing equations to calculate the director orientation of the liquid crystals. Then, using the director orientation, we derive the anisotropic properties of the liquid crystals.

## Results and discussion

Conventional liquid crystal devices typically consist of two electrodes and alignment layers, resembling a capacitor-like structure. However, in our model, we used a four-electrode liquid crystal device without any alignment layers, as shown in Fig. 3. Increasing the number of electrodes can create a more complex structure with higher reconfigurability. However, in this study, we chose to use four electrodes to demonstrate the concept with minimum computation and to analyze the results with less non-linearity.

The absence of fixed boundaries, such as alignment layers, allows for a greater degree of freedom in controlling the liquid crystal molecules using variable external forces. The control becomes more local rather than bulk, as there are no restricted degrees of freedom due to fixed alignment layers. This enables the liquid crystal molecules to be reconfigured in any direction in space and time, offering more flexibility and versatility in the manipulation of the liquid crystal properties.

There are several liquid crystal materials available in the nematic category, including 4-Cyano-4'-pentylbiphenyl (5CB), 4-n-octyl-4′-cyanobiphenyl (8CB), and 4-n-methoxybenzylidene-4'-n-butylaniline (MBBA), among others. In our simulations, we utilize 5CB liquid crystals with properties listed in Table 1. The simulated liquid crystal enclosure has dimensions of 30 × 30 × 50 μm3, as shown in Fig. 3.

**Table 1:** The4-Cyano-4'-pentylbiphenyl (5CB) Material properties, ${T}_{NI}=35.5^\circ{\rm C}$, where TNI is nematic-to-isotropic phase transition temperature.

Parameter	Symbol	Value
Viscosity	${\zeta }_{d}$	0.078 N·s/(m^2^)
S parameter	S	0.65
Elastic constant	L	2E − 11 N
Electric permittivity	${\varepsilon }_{\perp }$	6
	${\varepsilon }_{\parallel }$	18
Free space permittivity	${\varepsilon }_{0}$	8.85E − 12 F/m
Dielectric anisotropy	$\Delta \varepsilon$	12
Thermotropic constant	A	− 3.5E5 N/m^2^
	B	2.133E6 N/m^2^
	C	1.733E6 N/m^2^

For a twisted nematic LC cell, the response time^26^ is generally given as,

21 $$t=\frac{\gamma }{{\varepsilon }_{0}\Delta \varepsilon {E}^{2}}$$

The electric field, denoted by $E=V/d$, $V$ is the applied voltage, $d$ is the cell gap, $\gamma$ is the rotational viscosity of the liquid crystals. The first threshold voltage (~ 9 V for the simulated thickness) corresponds to the bend and splay transition, which has a lower value due to the geometry of the device. The second threshold voltage (~ 22 V) is for the twisting movement, where the orientation propagation occurs in a single direction perpendicular to the electrode surface.

**Figure 5:**
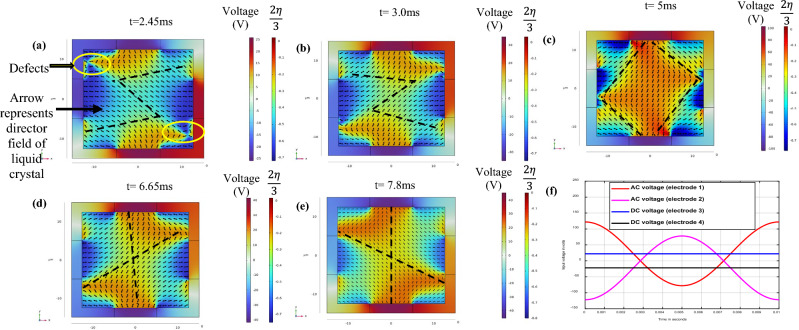
The transition in splay and bend phases to the voltage (Color plot) creates different patterns of director orientation in the XY plane, as given by the auxiliary variable in the X-direction* (*$\frac{2\eta }{3}$*)*. The director positions are depicted by arrow plots, and the director orientation is simulated by the applied voltages. (**a**,**b**) Show the dynamics of liquid crystal molecules creating Z and inverse Z patterns of molecular orientation in time and space for 2.4*5 ms and 3 ms, respectively.* (**d**,**e**) Depict the dynamics of liquid crystal molecules creating X patterns of molecular orientation orthogonal to each other in time and space for 6.65 ms* and 7.8 ms,* respectively. (**c**) Illustrates the dynamics of liquid crystal molecules creating rectangular patterns of molecular orientat*io*n in time and space for 5 ms*.* (**f**) Displays the applied voltages to the four different electrodes*.*

The simulations are conducted using an AC signal with a peak voltage of 125 V applied to electrodes 1 and 3, with different phases and offset voltages of + 22 V and − 22 V as shown in Fig. 5f, in order to introduce non-linearity and observe the reconfiguration of the director field and optical birefringence patterns. DC bias voltages of + 22 V and − 22 V are applied to electrode 2 and electrode 4, respectively, as shown in Fig. 3b. According to Eq. (21), the speed of molecular movement in a liquid crystal device is directly proportional to the applied voltage. In this case, it limits the driving voltage frequency to 100 Hz. The computation is carried out to calculate electric field and hydrodynamic response to that electric field. Electric field is changing with the applied AC voltage signal having a time period of 10 ms, and electric field for different voltage points are calculated quasi-statically. The hydrodynamic computation is carried out dynamically with a liquid crystal response time of 30 μs and the time resolution is 1 μs. The spatial resolution for the simulation is 1 μm for electric field computation and hydrodynamic study. For hydrodynamic simulation of liquid crystal, the response time for the liquid crystal directors is $10\,{\varvec{\mu}}{\varvec{s}}$, while the time period of the AC driving voltage is $10\,{\varvec{m}}{\varvec{s}}.$ The calculated liquid crystal response time is calculated $<=30\,{\varvec{\mu}}{\varvec{s}}$ using Eq. (21). For our hydrodynamic simulations, the liquid crystal directors rotate for a small angle compared to the full $\sim 90^\circ$ rotations in the conventional liquid crystal cells as the liquid crystal molecules are drive in continuous manner. Therefore, hydrodynamic simulations were carried out with the $30\,{\varvec{\mu}}{\varvec{s}}$ liquid crystal response time. The spatial resolution for 0.8 ${\varvec{\mu}}{\varvec{m}}$ for electric field computation and hydrodynamic simulation.

### Electrodynamic programming of liquid crystal molecules using DC bias voltage and AC voltage cycle

Creating splay bend phases or twisted nematic phases typically requires special patterning of the alignment layer. However, these patterns are not reconfigurable and have limited tunability due to the restricted degrees of freedom imposed by permanent alignment layers^27,28^. In this study, we demonstrate the generation of splay bend phases without the need for any special fabrication requirements, simply by using four electrodes. These phases can dynamically transition from one to another, with the phase changing its location in the XY plane over time. Twist deformations are formed in the XZ and YZ planes. This programming of the director field orientation in all planes is achieved through the superposition of electric fields that control the molecular orientation of the liquid crystals by generating different balancing torques on these molecules in space and time.

In conventional liquid crystal devices, the alignment layer is responsible for creating a permanent and uniform twisting moment across the cross-section. When an electric field is applied to such a device, the liquid crystal molecules align with the electric field, destroying the twisted molecular arrangement. In our device, on the other hand, the twisted nematic phase is shaped and controlled using both AC driving voltage and bias voltage, allowing for dynamic and flexible control of the liquid crystal orientation. In this study, different patterns evolving with time dependent electric fields are shown in a single configuration on AC and DC voltages to demonstrate the concept. Using this method different patterns can be created using different input voltage configurations.

By controlling the voltage configurations applied to the electrodes, it is possible to create and control different director field patterns with a thickness equal to the device thickness. One of the significant auxiliary variables, $\frac{2\upeta }{3}$, is used to compute the director field, as all the auxiliary variables are interdependent. Figure 5a,b, at 2.45 ms and 3 ms, the liquid crystal molecules create an Z-shaped and a inverted Z-shaped director field configuration, respectively. The observed defects in these configurations are due to the singularities created by the superposition of electric fields. The time steps used here are not exact inverse points to each other, as we maintain some offset to show small tunability in both shapes. In Fig. 5c, the director configuration changes to a rectangular shape at 5 ms. Figure 5d,e show that at 6.65 ms and 7.8 ms, the liquid crystal molecules create two X-shaped director field configurations orthogonal to each other. The director field patterns here can be reconfigured, tuned, and reoriented using different superpositions of electric field vectors. A small non-linearity in the sine function of the driving AC voltages creates some reconfigurable patterns. A more complex and nonlinear system will help to program more complex configurations.

In addition, we also control the nematic twisting of the liquid crystals in another plane, by utilizing the superposition of electric field vectors. In conventional twisted nematic liquid crystal cells, a permanent twisting structure is formed due to the alignment layers, and then the liquid crystal molecules are realigned using an electric field. A diffraction grating device using liquid crystals can create a beat structure with permanent photoaligned patterns^29^, these kind of patterns can be created for tunability and reconfigurability using current technique.

**Figure 6:**
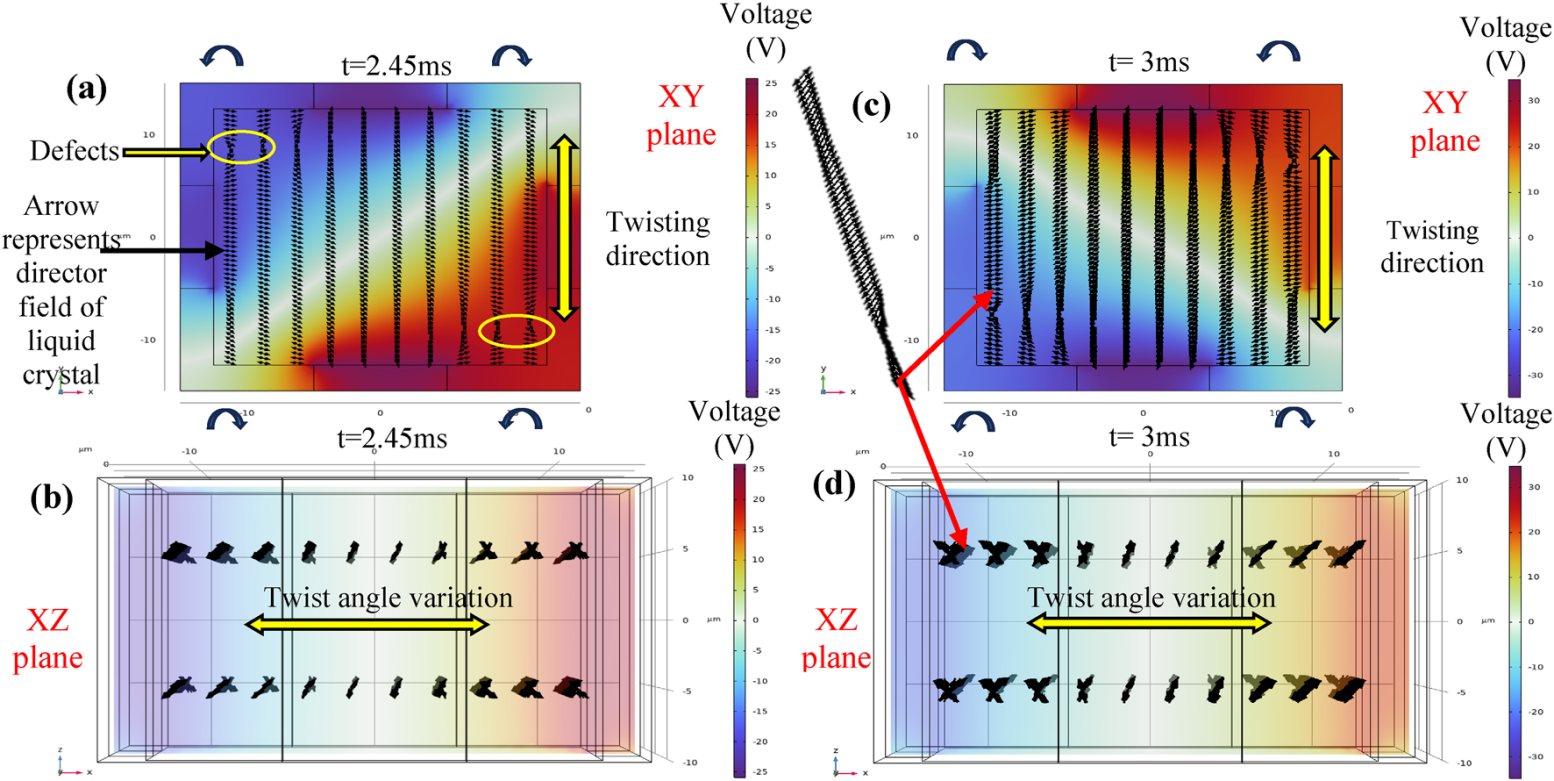
Dynamics of liquid crystal molecules in twisted nematic phase in space and time are shown in the following figures: (**a**) Twisting dynamics in XY plane at t = 2.45 ms, showing twisting in Y-direction. (**b**) Twisting dynamics in XZ plane at t = 2.45 ms, showing twist angle variation in X-direction. (**c**) Twisting dynamics in XY plane at t = 3 ms, showing twisting in Y-direction. (**d**) Twisting dynamics in XZ plane at t = 3 ms, showing twist angle variation in X-direction.

In our device, twisted nematic structures are formed through the superposition of electric field vectors. Furthermore, a beat structure is formed with increasing twist angles from the center to both ends in opposite directions. These twisted nematic patterns can be tuned and reconfigured. As shown in Fig. 6, we observed a twisting moment from one end of the device to the other end, indicating that we can control and change the chirality in space and time. Additionally, we observed distortions in the twist due to the presence of defects. Figure 6a and c show the twisting of liquid crystal molecules at 2.45 ms and 4.5 ms, respectively. Changes in applied voltages result in changes in torques due to the superposition of electric fields, leading to different patterns of twist movements. The twist angles are observed to change in space and time, depending on the applied field in both the XY plane directions. The change in twist angle with space and time is also observed along the Y-direction, as shown in Fig. 6b and d.

### Changing patterns of optical birefringence

**Figure 7:**
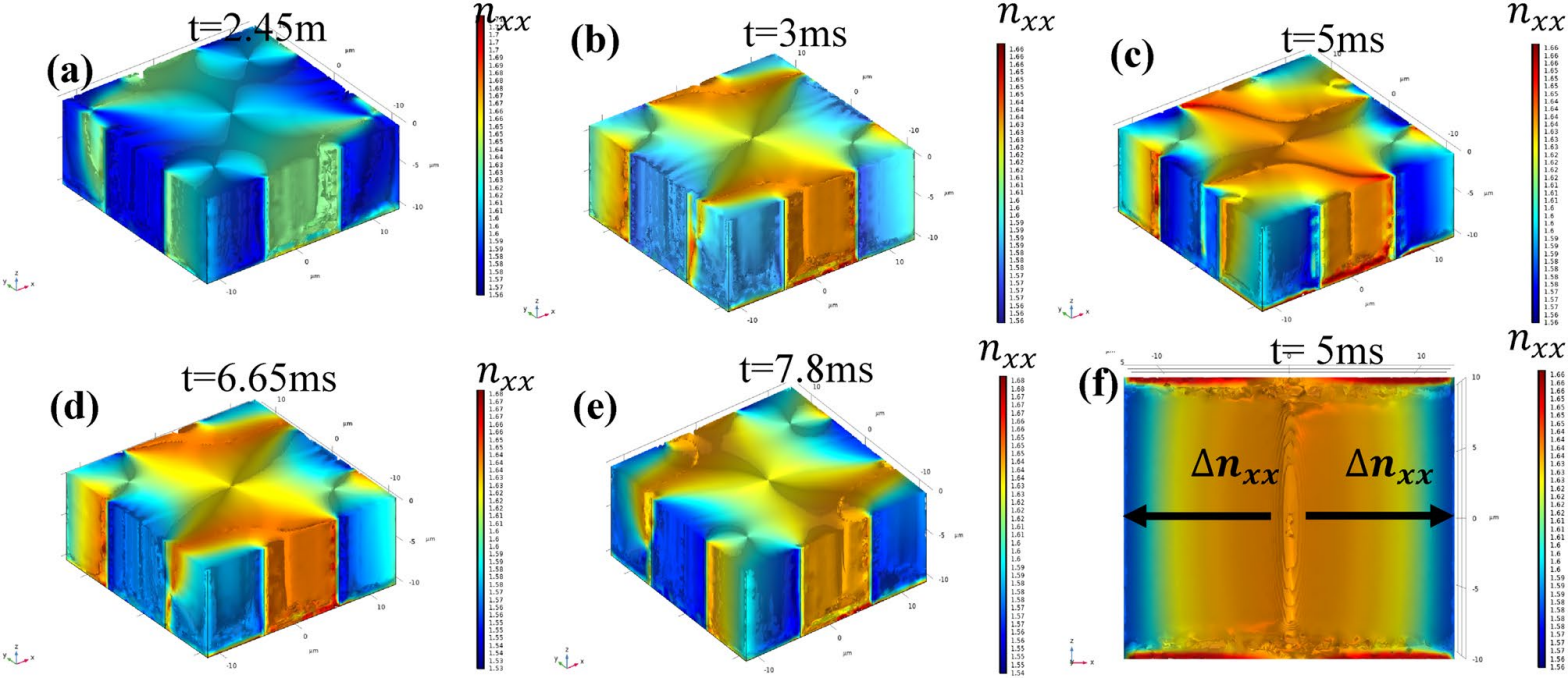
We derived patterns of optical birefringence using the director field patterns. (**a**,**b**) The dynamics of liquid crystal molecules create inverse Z and Z patterns of optical birefringence in time and space at 2.45 ms and 3 ms, respectively (cross-section in XY plane at Z = 0). (**d**,**e**) The dynamics of liquid crystal molecules create X patterns of optical birefringence orthogonal to each other in time and space at 6.65 ms and 7.8 ms, respectively (cross-section in XY plane at Z = 0). (**c**) The dynamics of liquid crystal molecules create rectangular patterns of optical birefringence in time and space at 5 ms. (**f**) Gradient of refractive index in the X direction is created due to twist angle variation along the Y direction in time and space at 5 ms (cross-section in XZ plane at Y = 0).

By controlling the director position, specifically the Euler angle and azimuthal angle of the liquid crystal orientation, using the superposition of electric fields, we can manipulate the anisotropic properties of liquid crystals in space and time. This allows us to create small domains with variable properties within a single domain by simply manipulating the applied voltages. In our study, we evaluated the changes in optical birefringence in the X, Y, and Z directions using local director fields. In the X and Y directions, the properties of the liquid crystals should be complementary to each other, as the voltage applied in the XY plane locally alters the major and minor axes of the liquid crystal molecules. However, in the Z direction, significant changes in the properties of the liquid crystals are not expected, as the patterns created have a thickness equal to that of the liquid crystal device. We calculated the optical birefringence of the liquid crystals using the director orientation of the liquid crystal molecules after electro-hydrodynamics computation (Fig. 7). The refractive indices in both the perpendicular and parallel directions were calculated for a wavelength of 632.8 nm, at different times and spatial locations. In the XY plane, the equivalent refractive index in the Y direction is complementary to the refractive index of the liquid crystals in the X direction.

The three-dimensional changes in the refractive indices are shown in Fig. 7a–f. The birefringence patterns created here replicate the director configuration patterns. Specifically, the birefringence patterns in Fig. 7a–e imitate the director field patterns from Fig. 5a–e in the XY plane. In the YZ plane, a gradient is created due to the twisting of liquid crystal molecules along the Y-axis. The variation of refractive indices in the Y-direction is a result of the twist movement of the liquid crystal molecules, as shown in Fig. 7f. This gradient structure can be changed in space and time.

## Conclusion

In this study, a new electric field vectors superposition technique was proposed to manipulate the orientation of liquid crystal molecules in space and time without the need for an alignment layer. Governing electro-hydrodynamics equations using auxiliary variables were proposed to simulate this concept. The simulations demonstrated three-dimensional control over splay, bend, and twist deformations in the liquid crystal domain using driving voltage and bias voltage, as revealed by the Q-tensor electro-hydrodynamic study. Results from the four-electrode simulations showed the potential application of this simulation program in modeling more complex and previously unseen three-dimensional and multi-electrode liquid crystal systems. The study also showed that replacing the alignment layer with bias voltage significantly improved the control over the liquid crystal director orientation through the superposition of electric field vectors. Furthermore, the three-dimensional four-electrode liquid crystal model demonstrated its ability to control birefringence patterns in space and time. The birefringence pattern in three-dimensional space was shown to be a result of bending and splaying in one plane and twisting in the third dimension, which changed dynamically with time and space. This demonstrated the programming of the liquid crystal directors to create different birefringence patterns dynamically. This new concept has the potential to improve the design of current liquid crystal devices and advance new applications in electro-optic, acoustic, and mechanical systems. It opens up possibilities for enhanced control and manipulation of liquid crystals in various technological applications.

## Data Availability

The datasets generated during and/or analyzed during the current study are available from the corresponding author on reasonable request.
